# EMT-related transcription factors and protein stabilization mechanisms involvement in cadherin switch of head and neck squamous cell carcinoma

**DOI:** 10.1016/j.yexcr.2022.113084

**Published:** 2022-02-25

**Authors:** Julia Ingruber, József Dudás, Dragana Savic, Gabrielle Schweigl, Teresa Bernadette Steinbichler, Maria do Carmo Greier, Matthias Santer, Sandro Carollo, Zlatko Trajanoski, Herbert Riechelmann

**Affiliations:** a Department of Otorhinolaryngology and Head and Neck Surgery, Medical University of Innsbruck, Innsbruck, Austria; b University Hospital of Tyrol, Innsbruck, Austria; c Laboratory for Experimental and Translational Research on Radiation Oncology (EXTRO-Lab), Department of Therapeutic Radiology and Oncology, Medical University of Innsbruck, Austria; d Tyrolean Cancer Research Institute, Innsbruck, Austria; e Institute of Bioinformatics, Medical University of Innsbruck, Austria

**Keywords:** Mesenchymal-to-epithelial transition, Cell-transfection, TissueFax, UPCI-SCC090, SCC25, FaDu

## Abstract

Epithelial to mesenchymal transition (EMT) describes a process where epithelial tumor cells acquire mesenchymal characteristics. EMT often correlates with invasion and an increased cell migration potential by losing cellular polarity and cell-cell junctions. It is mainly induced by tumor-microenvironment factors, such as TGF-beta 1 and IL-6, which activate the increased expression of the EMT-transcription factor (TF) Slug. We previously reported the Slug/Krüppel-like factor 4 (KLF4) switch in EMT in HNSCC, and found, that in human papilloma virus (HPV)-negative HNSCC Slug gene expression was significant higher represented, than in HPV- positive HNSCC.

The purpose of this study was to investigate the impact of KLF4 and Slug on the regulation of the cadherin switch and on the EMT phenotype.

Gene expression of KLF4 positive correlated with E-cadherin in 71 head and neck squamous cell carcinoma (HNSCC) patient tissue samples, which we also confirmed by the investigation of the Cancer Genome Atlas database (TCGA). HPV-transcripts contributed to stabilization of KLF4 at protein level, and simultaneously upregulated E-cadherin. Furthermore, ectopic KLF4 overexpression was associated with epithelial gene expression by induction of E-cadherin, β-catenin and 70-kDa heat shock protein (HSP-70). The presence of HSP-70 ensures the membranous localization of E-cadherin, therefore, the ability of cells to form cadherin/catenin complexes and cellular linkages.

In conclusion, KLF4 is a major regulator of the epithelial cadherin-adhesion in HNSCC.

## 1 Introduction

Loss of the epithelial markers E-cadherin and Cytokeratin and upregulation of mesenchymal proteins like Vimentin and N-cadherin are typical characteristics of epithelial – to – mesenchymal transdifferentiation (EMT). EMT allows primary epithelial tumor cells to acquire mesenchymal invasive phenotypes. However, EMT is often not fully exerted, but occurs only partial, leading to co-expression of epithelial and mesenchymal markers in the cancer cells [1–4]. According to our previous experimental data, EMT is a consequence of external stimuli from the tumor microenvironment, such as transforming-growth-factor-beta-1 (TGF-beta1) or Interleukin 6 (IL-6) [5], cellular metabolic stress and internal cell-events [1,6], which activate EMT-associated transcription factors as the members of SNAI gene family.

During development, EMT events occur as early as gastrulation and are frequent afterwards, for example in the delamination of the neural crest [7]. EMT consists of coordinated changes in cell-cell and cell-matrix interactions that lead to loss of epithelial features and acquisition of mesenchymal characteristics. A previous analysis has estimated that EMT changes the expression of about 4000 genes (10% of the human genome) [8], with two genes reproducibly changed in all forms of EMT: E-cadherin (downregulated) and Snail (upregulated). EMT is a major contributor to invasive and therapy resistant cells in head and neck squamous cell carcinoma (HNSCC). EMT is reversible, the mesenchymal tumor cell phenotype turns back to epithelial one, by a reverse process: mesenchymal-to-epithelial transition (MET) [9]. In this regard, both the partial nature of EMT and the return of the EMT process: MET, induces heterogeneity in the cancer cell nests resulting in cells at various levels of epithelial and mesenchymal differentiation [10].

In our previous published work, we observed a negative relationship between the pro EMT-transcription factor (EMT-TF) Slug and the epithelial anti EMT-TF KLF4. In contrast to Slug, KLF4 was found to be associated with the epithelial gene expression profile. Therefore, we reported the KLF4/Slug switch during EMT [5]. In EMT process, especially in case of TGF-beta1-induced EMT in the HNSCC cell line SCC-25, KLF4 protein levels decreased, whereas Slug and Vimentin increased.

It is well reported that the cadherin-catenin complex adhesion is the essential component of adherens junctions and the downregulation results in losing of cell-cell contacts, tissue instability and cancer invasion. Cadherin switch, which characterize the downshifting of E-cadherin and upregulation of N-cadherin leads tumor cells to become mobile and invasive by losing of their epithelial integrity [11,12]. Without dissemination, tumor metastases would not develop, but even bearing a high invasive potential, mesenchymal cells are unable to form metastases without re-gaining the epithelial characteristics [13–15], as they need to settle and *resume proliferation* at metastatic sites [1,16]. Clinically, both the EMT and the MET phases are essential in dissemination of tumor cells and expansion of the disseminated cells.

In 2010 it was published by Jennifer L. Yori et al. [17] that presence of KLF4 is sufficient for restoring of E-cadherin in metastatic tumor cells. Thereby, the metastasis suppressive role of KLF4 was described in promoting E-cadherin expression and preventing EMT in mammary epithelial cells. While multiple signals may potentially activate EMT, downregulation and even silencing of KLF4 were reported as the possible most relevant steps in induction of EMT, including the downshifting of E-cadherin at protein and mRNA level [17]. In addition, KLF4 was found to be associated with the MET phenotype, the ability to support cell proliferation and the (re)-activation of the epithelial adhesion package [17–19].

Here, we set up the hypothesis, that replacement of KLF4 with Slug and vice versa regulates the cadherin switch, which is essential for the regulation of invasive, proliferative/adhered forms of HNSCC tumor cells. In addition, based on scattered hints from the relevant previous literature [20], we hypothesize that HPV virus transcripts stabilize KLF4, which in consequence also stabilizes E-cadherin. Subsequently, we also hypothesize that in HPV-positive HNSCC: Slug is unstable, or it is not effective in the regulation of the cadherin adhesion. In several previous studies the members of the SNAI transcription factor family Snail (SNAI1) [21] and Slug (SNAI2) [22] are reported to downregulate E-cadherin. Moreover, Katafiasz et al. demonstrated that both, E-cadherin and N-cadherin are regulated by Slug oppositely. E-cadherin disappeared from the cell-surface of UM-SCC-38 cells, while mesenchymal N-cadherin was upregulated by Slug overexpression [23].

## 2 Materials and methods

### 2.1 Patients profile for gene expression analysis

In Supplementary Table S1 the study population is summarized. The tissue samples of 71 (for mRNA/gene expression analysis) and 58 (for quantitative immunohistochemical analysis) HNSCC patients (patient data listed in Supplementary Table S1) were surgically removed during pan-endoscopy following the patients ‘consent and in agreement with our ethic approval (UN4428, ethic commission meeting of 303/4,14, July 26, 2011), and used for RNA isolation, reverse transcription and real-time RT-PCR, or for immunohistochemical investigation respectively.

### 2.2 RNA extraction, reverse transcription and PCR

For RNA isolation 2–3 mm tissue slices were collected and lysed in 1 mL TRIzol® Reagent (Ambion®, Life technologies™, Carlsbad, CA, USA), and RNA was isolated following the instructions of the manufacturer. RNA concentrations were determined by absorption at 260 nm and by fluorometric measurements (Qubit, Invitrogen, Darmstadt, Germany), and RNA quality and integrity were identified by Qubit RNA IQ kit (Invitrogen). The proportion of intact RNA of total RNA isolates was at least 70%. Two micrograms of total RNA were reverse transcribed by M-MuLV Reverse Transcriptase with 2 μg of oligo dT_15_ (GeneON, Ludwigshafen am Rhein, Germany) in a ThermoQ heating and cooling block (Biozym, Hessisch Oldendorf, Germany). cDNA samples representing 10 ng original total RNA were subjected to real-time qPCR. The used primer sequences were downloaded from the PrimerBank of the Massachusetts General Hospital, Boston, MA, USA [24] and are listed in Supplementary Table S2. The primers were synthesized by Invitrogen, Darmstadt, Germany and were used together with the Sensifast Sybr Fluorescein Kit of Bioline (Labconsulting, Vienna, Austria) in a Bio-Rad MyiQ™ (Bio--Rad, Laboratories, Inc., Hercules, CA, USA) cycler according to the manufacturer’s protocol.

GAPDH was used as housekeeping gene, and relative quantities of CDH1, CTNNB1, CDH2, HSP70, KLF4, SNAI2 transcripts were calculated by pair-wise differences of threshold cycles (Δ_CT_) of gene of interest and the loading control housekeeping gene [25]. According to Livak and Schmittgen, in the final analysis, we used the relative quantification and related the PCR signal in both HNSCC and control mucosa to a reference, which was the mean value of the control samples [5]. The identity of the PCR products of genes discussed in this study were confirmed by Sanger sequencing by Microsynth Austria (Vienna, Austria).

### 2.3 Gene expression data

Gene expression data from The Cancer Genome Atlas (TCGA)’s Head and Neck Squamous Cell Carcinoma (HNSCC) cohort (n = 501 samples) were downloaded from the National Cancer Institute’s Genomic Data Commons (GDC) (https://portal.gdc.cancer.gov/). The gene-level expression data were then converted to log (TPM + 1) (TPM= Transcripts Per kilobase Million) values, for downstream analysis and visualization purposes.

The TCGA codes were associated to 3 different sample type: metastatic (n = 2 samples), primary tumor (n = 482 samples) and normal solid tissue (n = 17 samples), and therefore three different gene expression subgroups were generated. Finally, in order to obtain the final output, the 3 gene expression datasets were filtered based on the patient’s UUID identifier and the list of relevant genes.

### 2.4 KLF4 mutation analysis

The whole protein coding region of KLF4 (Sequence ID: NM_001314052.2) was amplified in HNSCC tissue- and FaDu cells -derived cDNA samples using the primers forward: ATGAGGCAGCCACCTGGCGA; reverse: TTAAAAATGCCTCTTCATGTG and Go-Taq Hot Start master mix (Promega, Madison, WI, USA). The resulting 1541 base pairs PCR product was electrophoresed in a 1% agarose TAE gel, excised on a transilluminator, and the PCR product DNA was purified using the Minelute DNA elution kit of Qiagen (Hilden, Germany). The purified DNA product was sent to Microsynth (Vienna, Austria), which performed Sanger sequencing from both forward and reverse primer directions. The received sequences were submitted to sequence alignment analysis using NCBI Blast (National Center of Biotechnology Information, Bethesda, MD, USA).

### 2.5 Immunofluorescence, immunohistochemistry and TissueFax

Indirect immunofluorescence staining for Slug, KLF4, Cytokeratin and E-cadherin was performed on a Ventana Discovery Classic Immunostainer (Ventana, Tucson, AZ, USA). Primary rabbit monoclonal antibodies for Slug and KLF4, and pre-diluted mouse monoclonal E-cadherin and Cytokeratin antibodies were used and diluted as suggested by the manufacturers (Table 1). Secondary anti-rabbit IgG conjugated with Alexa Fluor™ 594 (cat. Nr. A21207) and anti-mouse IgG1 conjugated with Alexa FluorTM 488 (cat. Nr: A21121) or anti-mouse IgG2a conjugated with Alexa FluorTM 555 (cat. Nr. A21137) were used for the detection of the primary antibodies; were purchased from Molecular Probes (Eugene, Oregon, USA) and used at 1:200 final dilutions. Cell nuclei were counterstained by DAPI, purchased from Molecular Probes. Isotype control stainings were done using isotype matching mouse and rabbit IgG antibodies as listed in Table 1. Autofluorescence reaction was limited by the TrueView™ autofluorescence quenching kit and all slides were covered with Vectashield Vibrance (both from Vector Laboratories, Burlingame, CA, USA). Immunofluorescence intensities were acquired and photographed by TissueFaxs system (TissueGnostics, Vienna, Austria), using a PCO Pixelfly CCD monochrome camera (PCO Inc., Kelheim, Germany) [26].

Enzyme immunohistochemistry was carried out on formalin-fixed, paraffin-embedded tumor tissue sections for HSP-70 and KLF4, using antibodies as follows: mouse monoclonal to HSP-70, and rabbit monoclonal to KLF4 (further details in Table 1). The immunoreactivity was developed by universal (mouse/rabbit reactive) secondary antibody and the DAB Map detection kit of Ventana. The immunohistochemistry staining reactions were acquired in TissueFaxs brightfield system using a Pixelink camera (Pixelink, Rochester, NY, USA). The acquired images were transferred and quantified in HistoQuest 7.143 software (TissuG-nostics, Vienna, Austria). DAB staining was recognized using a singlereference-shade colour deconvolution algorithm [27]. Evaluation of staining mean intensities and of the percentage ratio of positive cells relative to all cells were further processed based on the DAB-staining above the threshold of the negative control. All tissue sections were acquired and quantified using the same defined template. Cut-off values were set based on staining intensity of isotype control immunoglobulins, the positive and negative reactions were confirmed by the backward connection function of the HistoQuest 7.143 software.

## 3 Optical intensity measurements on immunfluorescence images

Representative immunfluorescent reactions for E-cadherin (green, Alexa Fluor 488), KLF4 (red, Alexa Fluor 594) and Slug (red, Alexa Fluor 594) immunofluorescence signals were quantified using TissueQuest. Tumor cells of typical structured relatively homogenous cancer cell nests were segmented by the DAPI counterstaining using the TissueQuest software (Tissuegnostics). The cell nuclei were recognized by their size and their minimum fluorescence intensity according to instructions of the software provider and previous publications [5,26,27]. Cell specific immunofluorescence signals for E-cadherin were determined taking the cellular radius from the nuclei into consideration in the cell surface using growing algorithm outside of the cell nuclei. For both KLF4 and Slug the fluorescence signal was determined inside the nuclei. The typical “circle-formed” cancer cell nest was put to the middle of the scale lines. The following areas were labelled and evaluated as regions of interest: internal 50 μmeter radius core circle, and the following ring-form regions of interest: 50–80 μmeter, 80–110 μmeter, 110–140 μmeter, 140–170 μmeter, 170–200 μmeter. The regions of interests contained 300–500 cells, and the immunofluorescence signal intensity of E-cadherin, KLF4 and Slug was determined in all cells of the regions of interest. The mean immunofluorescence signal, the standard deviation and the number of analyzed cells were used for the plots.

### 3.1 Cell lines and treatment conditions

Three HNSCC cell lines, namely SCC25, UPCI-SCC090 (both from the German Collection of Microorganisms and Cell Cultures, DSMZ, Braunschweig, Germany) and FaDu (American Type Culture Collection (ATCC), Manassas, VA, USA) were used in this study. The cells were cultured in three different media: i) SCC-25 cells in DMEM/Ham’s F12, with 10% FBS ii) UPCI-SCC090 cells in EMEM Medium supplemented with 10% FBS, 2 mM l-glutamine, 100 units/ml penicillin and 100 μg/mL streptomycin, and iii) FaDu cells were given Minimum Essential Medium (Eagle) with Earle’s BSS (Merck Millipore, Vienna, Austria). The last culture medium was supplemented with 2 mM L-glutamine, 1,5 g/L sodium bicarbonate, 0.1 mM non-essential amino acids (Gibco, Life Technologies, Grand Island, NY, USA), 100 U/mL penicillin, 100 μg/mL streptomycin, and 10% fetal calf serum (Merck Millipore, Vienna, Austria). For experimental purposes all cells were cultured in an albumin-containing corresponding culture medium where serum proteins were replaced by 4.4 g/L bovine serum albumin from Serva (Heidelberg, Germany). In particular, cells were screened regularly for mycoplasma contamination using a Mycoplasma PCR detection kit (cat. Nr. G238, Applied Biological Material, THP, Vienna, Austria).

For the treatment with 50 ng/mL IL-6 [28] or with 1 ng/mL TGF-beta1 [29] (RnD Systems, Minneapolis, MN, USA), 5 × 10^4^ cells/mL were plated on 6-well cell culture plates (BD Falcon, Vienna, Austria) in 3 mL albumin-containing medium per well, supplemented with the treatment factors and cultured for 72 h, followed by replacement of the medium and treatments for an additional 48 h [5]. After the end of the treatment procedure, cells of the wells (one well – one treatment sample) were scraped into 500 μL of RIPA buffer (50 mM Tris HCl/pH:7.4, 1 mM EDTA, 0.5 mM EGTA, 1% Triton X-100, 0.25% sodium deoxycholate, 0.1% sodium dodecylsulfate, 150 mM NaCl, 10 mM NaF, 1 mM PMSF; 10 μL proteinase inhibitors of Invitrogen “Halt Inhibitors mix”/mL RIPA buffer)/culture dish or into 1 ml Trizol (Invitrogen, Darmstadt, Germany). For protein isolation the cell suspension was vortexed and incubated 3-times for 15 min on ice, homogenized in22G needles and centrifuged at 15,000× g for 15 min at 4 °C. For RNA isolation cells in Trizol were homogenized by pulling through three times in 22G injection needles followed by the Trizol RNA isolation procedure as described by Invitrogen. Reverse transcription and real-time qPCR were done as described above.

### 3.2 Ectopic overexpression of slug and KLF4

SCC-25, UPCI-SCC090 and FaDu cells were transiently transfected with open reading frame (ORF)-containing expression vectors purchased from SinoBiological (Beijing, China), where the protein coding sequence of Slug (Cat. Nr. HG11196-UT) or KLF4 (Cat. Nr. HG12321-UT) was controlled under the CMV promoter in a pCMV3 plasmid construct. For control transfections the negative control vector (Cat. Nr: CV011) was used [“empty vector controls”]. The purchased expression vectors were amplified in Oneshot competent bacteria (Invitrogen, Darmstadt, Germany), and the plasmids were isolated using the Purelink Expi endotoxin-free plasmid purification kit (Invitrogen). 10^5^ cells/ml medium were plated in 6-well plates (3 ml cell suspension/well) for transfection with ORF-plasmids. The transfection was done on the following day using 4 μl Viafect (Promega, Madison, WI, USA): 2 μg plasmid per well, in 2:1 ratio and following the instructions of the manufacturer. The transfection was repeated 48 h after plating. Subsequently cells were harvested 48 h after the second transfection and used for protein and RNA isolation.

### 3.3 Western blot analysis of the protein expression of HSP-70, E-cadherin, N-Cadherin, KLF4 and slug in HNSCC cell lines

Total proteins were isolated from original and transfected SCC25, UPCI-SCC090 and FaDu cells. Therefore, 1 ml of RIPA lysis buffer (50 mM Tris HCl/pH:7.4, 1 mM EDTA, 0.5 mM EGTA, 1% Triton X-100, 0.25% sodium deoxycholate, 0.1% sodium dodecylsulfate, 150 mM NaCl, 10 mM NaF, 1 mM PMSF) was mixed with 10 μl HALT proteinase inhibitors of Invitrogen. Cells were vortexed and incubated 3-times on ice (15 min), homogenized in 22G needles and centrifuged at 15,000 g, 15 min, 4 ° C. The supernatant was saved, and the protein concentration measured by the Pierce 660 nm protein assay (Pierce, Rochford, IL, USA), following the description of the manufacturer. Next, 10 μg protein from all samples was further processed for western blot, using Invitrogen NuPage gels, electrophoresis and blotting system. Western blot detection was performed following a previously published protocol [5,30]. The primary antibodies used in the experiments were the following: rabbit monoclonal anti-KLF4 and rabbit monoclonal anti-Slug, anti--N-Cadherin, anti-E-cadherin, anti-ß-Catenin, anti-HSP-70, and anti-GAPDH was used for detecting the loading control. Antibodies were diluted und used in conditions according to manufacturer recommendations (further details in Table 2). For the specific signal detection near infrared fluorescence conjugates labelled anti-rabbit IgG and various anti-mouse IgG secondary antibodies were used, both available from LiCor Bioscience (Li-Cor Biosciences, Bad Homburg, Germany) and Azure (Azure Biosystems Central Europe, Prague, Czech Republic). For low abundant proteins as Slug and KLF4: chemiluminescence detection system using peroxidase labelled anti-mouse or anti-rabbit secondary antibodies were needed and peroxidase substrate of Radiance Plus Kit (Azure) was used. An Azure C500 documentation system was used to visualize the protein blots. The optical density (OD) of western blot bands was measured by using the Li-cor Image Studio Lite software [5]. The ODs of proteins of interest were normalized by the ODs of GAPDH. The normalized ODs were related to the ODs of the control samples in the experimental set. The distribution of the relative normalized ODs of E-cadherin, β-catenin and HSP-70, was statistically tested by the DAgostino and Pearson omnibus normality test. The differences in the groups (control, empty vector transfected, Slug and KLF4 plasmid transfected; as well as TGF-beta1 or IL-6-treated) were compared by parametric or non-parametric ANOVA, followed by pairwise comparisons with the control data set by Mann-Whitney *U* test or unpaired Students *t*-test depending on the normal or non-parametric distribution of the data sets.

### 3.4 Statistical analysis

Distribution of data sets was tested by D’Agostino and Pearson omnibus normality test. Means of normal distributed datasets were compared using parametric tests and medians of non-parametric distributed datasets were compared using corresponding nonparametric tests. Correlations were tested with Spearman r. In all cases confidence interval was set to 95%. Statistical significance was claimed at p = 0.05.

## 4 Results

### 4.1 KLF4, E-cadherin and slug gene expression analysis in HNSCC patient samples

KLF4 acts as a transcriptional negative regulator of genes critical for EMT and potently induced E-cadherin mRNA and other epithelial biomarkers as well [19,31]. Our previous data revealed a negative correlation between KLF4 and Slug gene expression, analyzed in 37 HNSCC tissue samples [5].

**Fig. 1:**
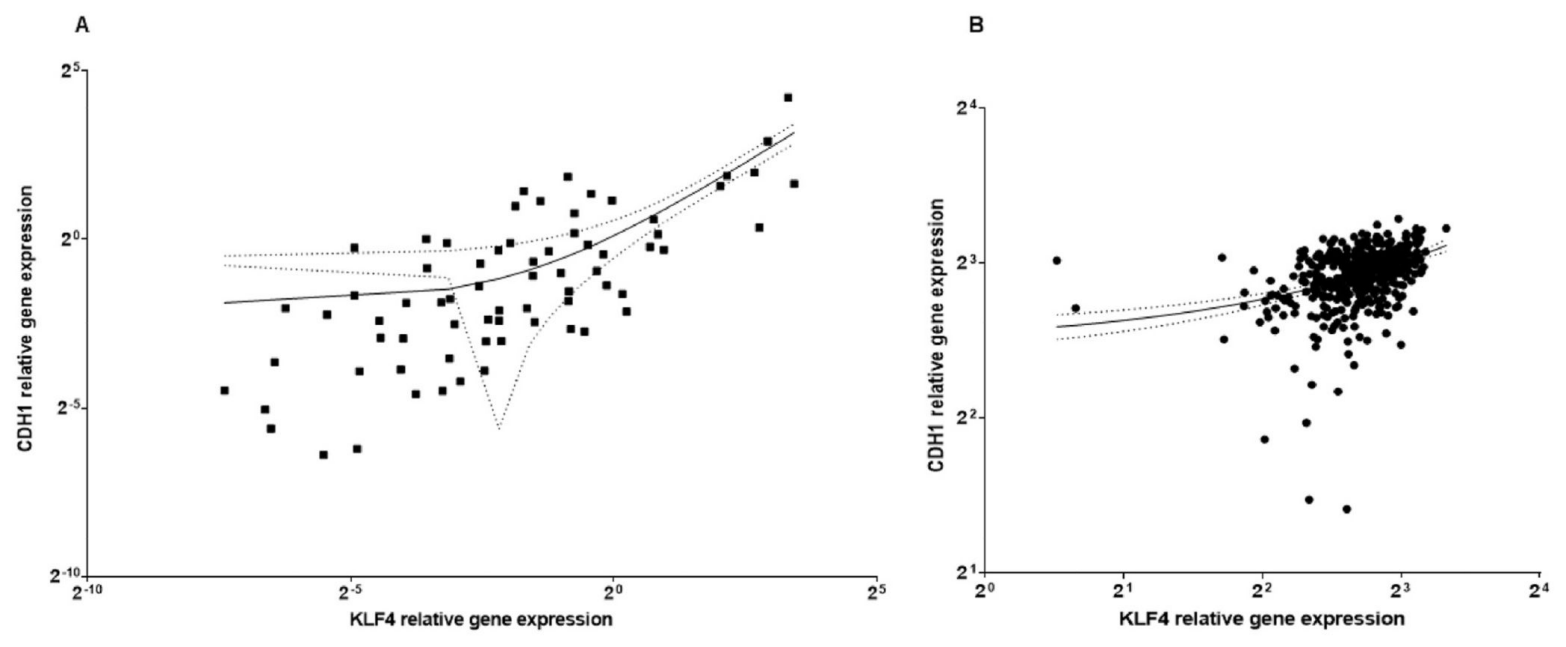
A) Relative gene expression related to reference value of normal mucosa of E-cadherin (CDH1) and KLF4 analyzed in 71 HNSCC samples. B) Gene expression in log (TPM + 1) values of KLF4 and CDH1 (E-cadherin) in the TCGA database. At mRNA level KLF4 and CDH1 (E-cadherin) showed significant positive correlation, both in our own clinical RNA data bank N = 71 (A), and in TCGA, N = 482 (B). Corresponding regression lines at 95% confidence interval.

To determine causal relationships between KLF4, E-cadherin and the EMT-associated transcription factor Slug, 71 HNSCC patient tissue samples were included for gene expression analysis. We identified a significant positive correlation of KLF4 and E-cadherin gene expression (Spearman r: 0.7, p<10-^4^) in our HNSCC RNA data bank (Fig. 1 A), as well as in the HNSCC data bank of The Cancer Genome Atlas (TCGA) based on 482 primary HNSCC patients (Spearman r: 0.4, p<10-^3^) (Fig. 1 B).

**Fig. 2:**
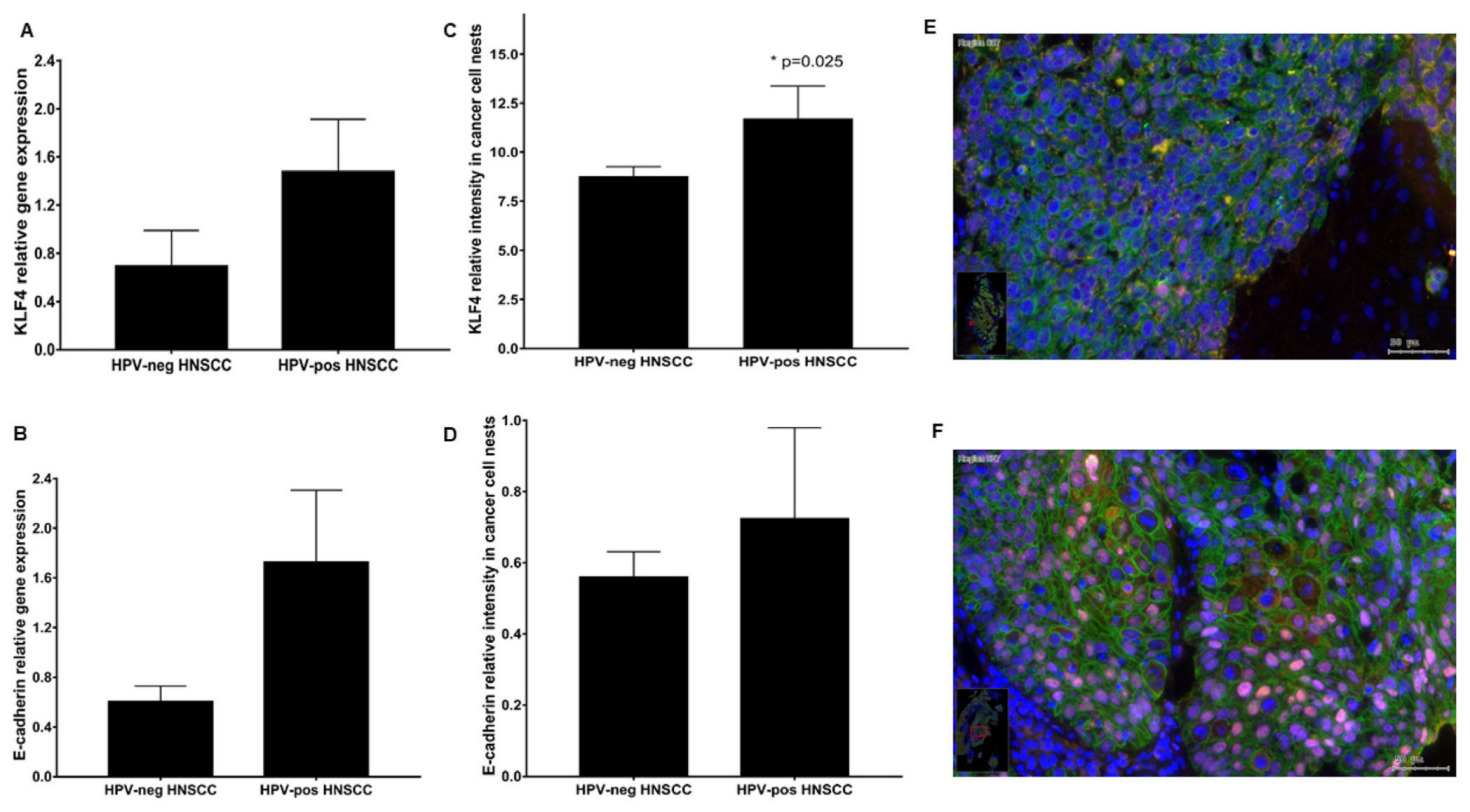
Relative gene expression of KLF4 (A) and E-cadherin (B) in HPV-negative and in HPV-positive HNSCC tissue samples. The relative staining intensity of KLF4 (C) was significant higher in HPV-positive HNSCC than in HPV-negative HNSCC. E-cadherin staining intensity related to the mean of control epithelium was increased in HPV-positive HNSCC (D), but the difference to HPV-negative HNSCC was not significant. Immunofluorescence labelling in HPV-negative (E) and HPV-positive (F) oropharynx carcinoma with KLF4 (pink) and E-cadherin (green) combination (E–F). In the HNSCC without HPV genetical background a diffuse localized E-cadherin staining reaction was detected, and only a few nuclei in the cancer cells nest contained a pink KLF4 reaction (E). In HPV-positive HNSCC tissue samples a strong KLF4 positive nuclear staining reaction combined with membranous E-cadherin expression were detected (F). Bars: 50 μmeter. Case numbers included: A-B: N = 70; HPV-: 36, HPV+:34; C: N = 58; HPV-negative: 45, HPV-positive: 13, D: N = 42, HPV^–^ :32, HPV^+^:10).

As a next step, gene expression of KLF4 and E-cadherin was further analyzed in mRNA samples isolated from patients with and without HPV background. HPV-related HNSCC tissue samples were selected by immunostaining of the surrogate biomarker p16^INK4^. Tumor biopsies were considered p16 positive if 70% or more of tumor cells expressed p16 [32]. HPV DNA PCR analysis was used as reference method: the sensitivity of p16 was 78% and the specificity was 79% [33]. Lower Slug gene expression in HPV-positive HNSCC was already confirmed by our group and published elsewhere [5]. Tendentially, HPV-positive HNSCC showed an increased KLF4 gene expression (Fig. 2 A), but this difference to HPV-negative samples was not statistically significant (N = 70; HPV-: 36, HPV+:34; p = 0.23). Similarly, in HPV-positive HNSCC E-cadherin gene expression was higher than in HPV-negative HNSCC (Fig. 2 B), but it also did not show a significant difference at 95% confidence interval (N = 70; HPV-: 36, HPV+:34; p = 0.09).

In a further step, we examined the HPV-product stabilization effect of KLF4 at protein level in paraffin embedded tumor tissue samples and three HNSCC cell lines.

### 4.2 Protein level analysis of KLF4 and E-cadherin in relation to HPV genetical background in tumor tissue and HNSCC cell lines

Gunasekharan et al. reported in 2016 that KLF4 protein levels were increased in HPV-positive cells through suppression of cellular miR-145, and due to post-translational regulations [20]. Based on this knowledge, we were also interested in the protein level differences of KLF4, and in consequence of E-cadherin in HPV-positive and negative HNSCC. At protein level both, percentage of cells (p = 0.04) (Supplementary Figure S1 A) and staining intensity of KLF4 (p = 0.025) (N = 58; HPV-negative: 45, HPV-positive: 13) were higher in HPV-positive than in HPV-negative primary HNSCC (Fig. 2 C). We could confirm the data of Gunasekharan et al. As HPV virus products contribute to the stabilization effect on KLF4, it was reasonable to test if the KLF4-regulated cell-adhesion molecule E-cadherin protein levels are also increased in HPV-positive HNSCC.

Similarly, to the gene expression analysis staining intensity of E-cadherin related to the mean of control epithelium was higher in HPV-positive HNSCC (p = 0.5), but the difference to HPV-negative tumor samples was not significant (Fig. 2 D). Tendentially lower E-cadherin positive staining intensity was observed in HPV-negative HNSCC tissue samples (N = 42, HPV :32, HPV^+^:10, Mann-Whitney test).

The evaluated percentage of cells, stained with membranous E-cadherin (Supplementary Figure S1 B) were similar in both groups, HPV-positive and HPV-negative HNSCC.

The post-transcriptional regulation and stabilization effect of KLF4 by HPV-products could be demonstrated in HPV-positive HNSCC tissue samples. According to the immunohistochemical evaluation, KLF4 was differently expressed in HPV-positive and HPV-negative HNSCC tissue samples. In HPV-negative oropharynx carcinoma KLF4 was low represented in most of all tumor cell nuclei, together with a diffuse localized membranous E-cadherin reaction (Fig. 2 E). In HPV-positive tumor tissue samples a strong KLF4 positive nuclear staining reaction all over the cancer cell nest combined with a membranous E-cadherin reaction was detected (Fig. 2 F).

**Fig. 3:**
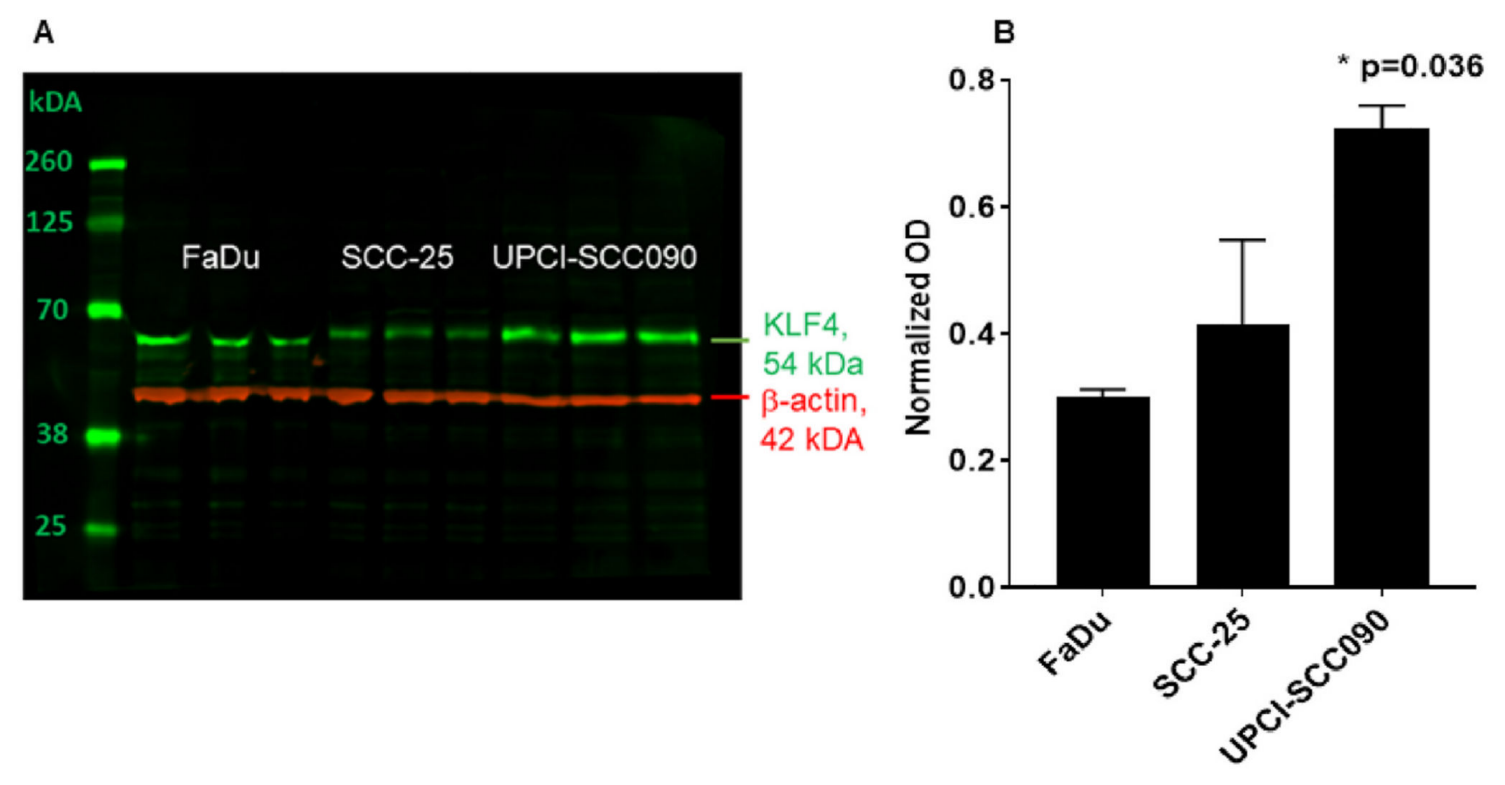
A) KLF4 protein (green) was analyzed by western blot detection in three repeats. Beta-actin (red) was used as loading control in samples of three HNSCC model cell lines: FaDu, SCC-25 and UPCI-SCC90. KLF4 protein was present in untreated conditions in all 3 cell lines examined. Both SCC-25 cells and FaDu cells had significantly lower KLF4 levels than UPCI-SCC090, which letter cells showed a stabilized and constitutively high KLF4 protein expression. The optical density of the specific bands of interest was normalized with that of beta-actin. B) Optical density based quantification of KLF4 in 3 HNSCC cell lines with significantly increased KLF4 expression in the cell line UPCI-SCC090 (p= 0.036 by Kruskal-Wallis test). The three repeats of the three cell lines presented in Figure A were used for the quantification in Figure B.

Three HNSCC cell lines: SCC25, FaDu and UPCI-SCC090 were used to determine the constitutive KLF4 protein levels in untreated conditions. FaDu cells became confluent at day 5 of culture, SCC25 and UPCI-SCC090 at day 8–10 (Supplementary Figure S3). As shown in Fig. 3, SCC-25 and FaDu cells (Fig. 3 A) had significantly lower KLF4 protein levels than UPCI-SCC090 cells (Fig. 3 B, N = 3; p = 0.036 by Kruskal-Wallis-test). In FaDu cells a minor difference in the electrophoretic condition of KLF4-band was observed, which was not due to mutations in the protein-coding sequence (as proved by Sanger sequencing).

In accordance to our previously described results, we confirmed the stabilized KLF4 protein levels detected by immunohistochemistry in HNSCC tumor samples now also by a complementary methodology using western blot in an HPV-positive cell line: UPCI-SCC090 (Fig. 3 B).

### 4.3 Localization and correlation of KLF4, E-cadherin and slug in HNSCC tumor tissue samples

In a further step, the relationship of KLF4, E-cadherin and Slug, were investigated immunohistochemically in 58 HNSCC patient tissue samples. All proteins were detectable in immunofluorescence and additionally interpreted and evaluated by TissueFaxs. We investigated the correlation of KLF4 and E-cadherin staining intensity of HNSCC relative to the mean intensity level of normal control mucosa surgically obtained by uvulopalatopharyngoplasty (UPPP) used as reference value. Although, it was not possible to find a significant positive correlation between KLF4 and E-cadherin in HNSCC (p = 0.2393), the tendency was similar to the results of mRNA gene expression analysis. In contrast, we observed a negative correlation between Slug and E-cadherin relative staining intensity, which was at border of significance (p = 0.05); (Supplementary Figure S2).

**Fig. 4:**
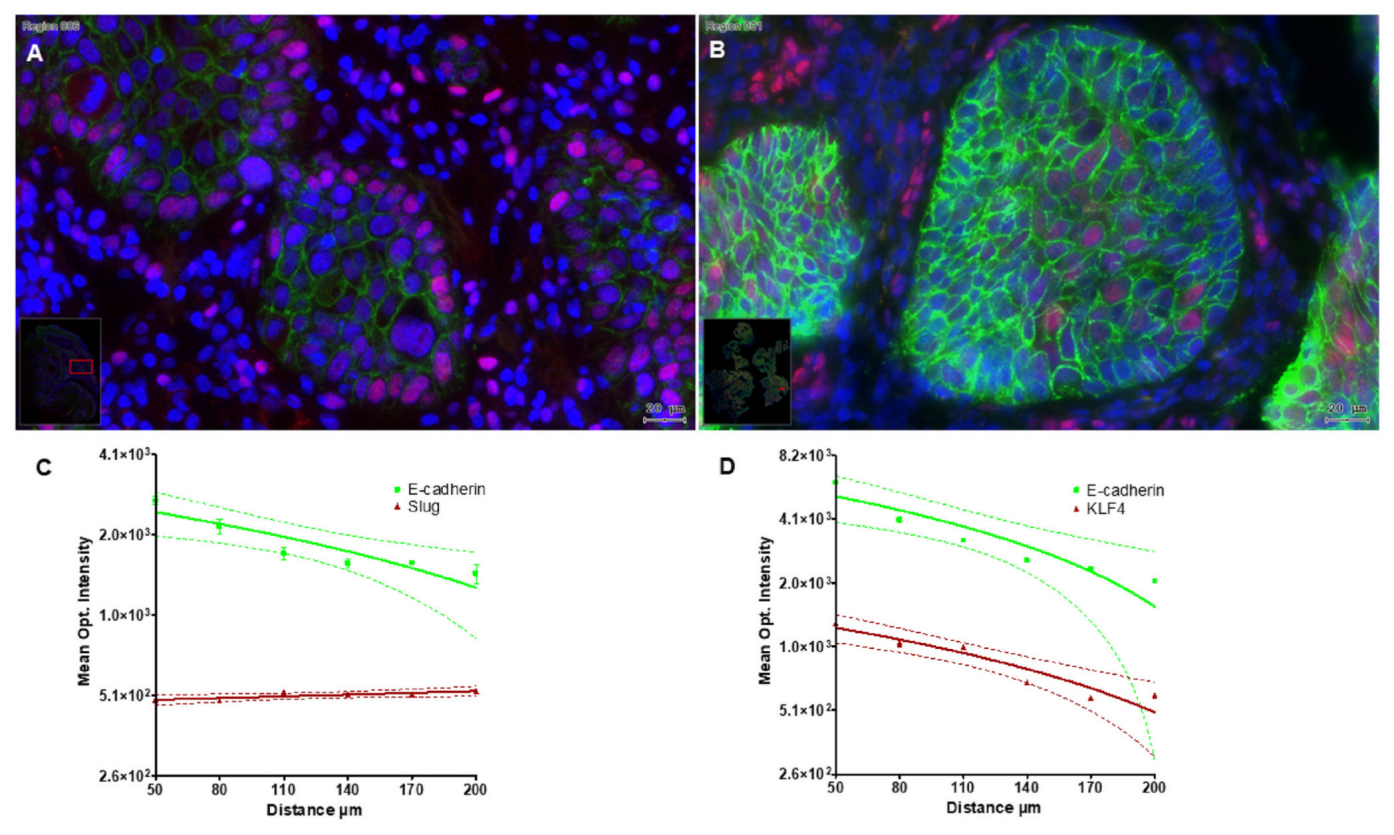
HPV negative HNSCC tissue sample: Figure A represents Slug positive tumor cells (pink) at the external part of the cancer cell nest and a decreasing gradient of E-cadherin (green) from the middle of the cancer cell nest towards to the border area. B) Comparison of immunofluorescence labelling of KLF4 (pink) and E-cadherin (green). KLF4 showed a positive staining reaction in the middle of the cancer cell nest, at the same time, it got lost in the cell nuclei of the tumor cells at the border region. The epithelial E-cadherin staining intensity reduces from the centre of the cancer cell nest toward to the leading edge and reveal the structure of the tumor node. The quantified regions of interest contained 300–500 cells. Bars: 20 μmeter. The changes of the immunofluorescence optical intensity depended of the distance from the middle of the cancer cell nest were plotted in (C) for E-cadherin and Slug, and in (D) for E-cadherin and KLF4.

As the next step we investigated the spatial distribution of Slug, KLF4 and E-cadherin in HNSCC tissue samples. The presented Fig. 4 displays an inverse gradient of localization between KLF4 and E-cadherin versus Slug, which is the manifestation of the cellular heterogeneity in the cancer cell nest on response to the stroma microenvironment. Similar observation has been published by Oshimori et al. in 2015 [10].The cells in the external part of the cancer cell nest, located at the tumor-stroma-interface bestow mesenchymal properties with high Slug (Fig. 4 A) and low E-cadherin and KLF4 expression, whereas, the cells in the middle of the cancer cell nest retain the epithelial E-cadherin/KLF4 expression (Fig. 4 B). These changes were also quantified in typical cancer cell nests as displayed in Fig. 4 C-D. Mean optical intensities of E-cadherin and KLF4 continuously decreased from the middle of the cancer cell nest towards the border (Fig. 4 C-D), whereas slug intensities slightly increased in the same direction (Figure C). According to Oshimori et al. these tumor-stroma-interface EMT cells gain increased protection against anti-cancer drugs, reprogram antioxidant metabolism and are supposed to be the major components in tumor recurrence.

### 4.4 TGF-beta1 was not able to downregulate E-cadherin mRNA in the HPV-positive cell line UPCI-SCC090

In patient histological tissue samples the epithelial markers as Cytokeratin, E-cadherin and KLF4 got decreased towards to the border of the cancer cell nests. Stroma-derived cytokines like IL-6 and environmental growth factors as TGF-beta1 downregulate E-cadherin [11,34], and modify the protein levels of EMT-related transcription factors. Pro EMT-TFs like members of the SNAI gene family increase, whereas KLF4, as anti-EMT-TF shows a reduction at mRNA and protein [5,27].

To study these effects more closely, mRNA gene expression and western blot analysis of E-cadherin, N-cadherin and HSP-70 proteins were performed in control and 1 ng/ml TGF-beta1 and in 50 ng/ml IL-6 treated conditions in FaDu, SCC-25 and UPCI-SCC090 cell lines. HSP-70 is required for the stabilization and cell membrane localization of the E-cadherin-β-catenin complex [35]. Therefore, changes in HSP-70 expression also significantly influence the cadherin switch.

**Fig. 5:**
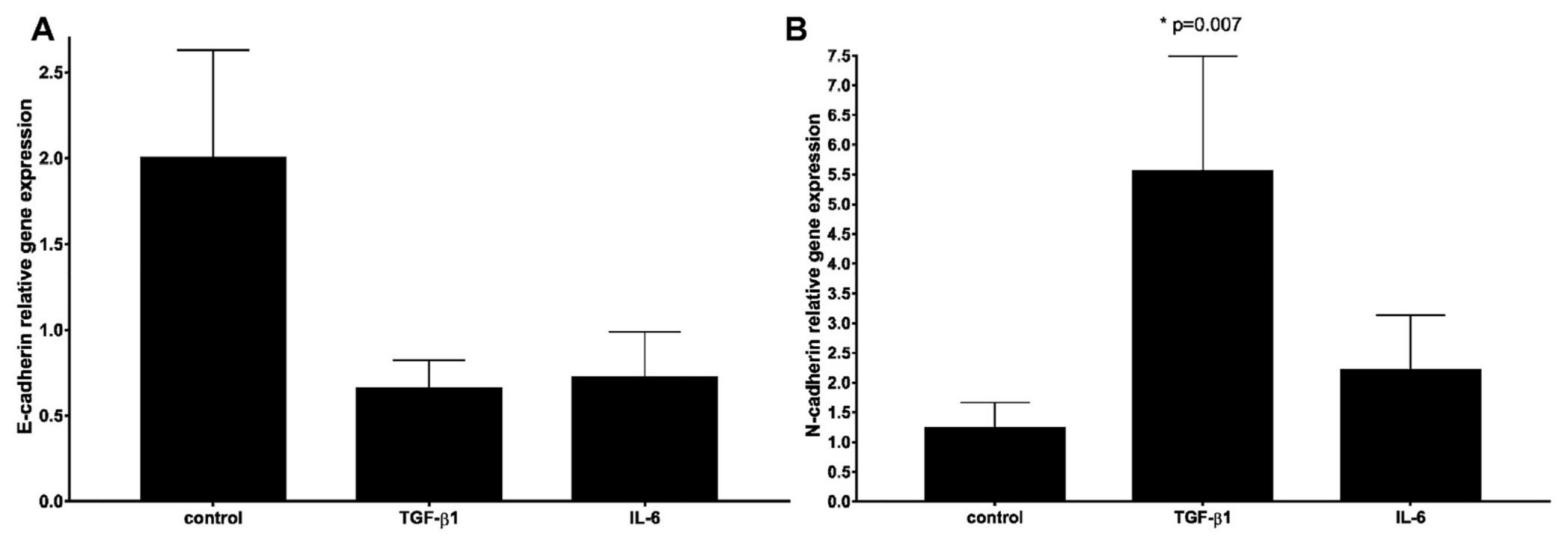
Cadherin switch induced by TGF-beta1. Relative gene expression of E-cadherin (A, N = 6) and N-cadherin (B, N = 8) in SCC-25 cells after treatment with 1 ng/ml TGF-beta1 or 50 ng/ml IL-6.TGF-beta1 and IL-6 induced visible but not significant decrease of E-cadherin and TGF-beta1 induced a significant increase of N-cadherin.

The changes in the gene expression of E-cadherin, N-cadherin and HSP-70 as a result of TGF-beta1 and IL-6 treatment were examined by relative gene expression analysis. In SCC-25 cells TGF-beta1 and IL-6 induced visible, but statistically not significant downshifting of E-cadherin gene expression (Fig. 5 A). Similar changes were observed in UPCI-SCC090 cells (Supplementary Figure S4 C) and in FaDu cells (Supplementary Figure S4 F). N-cadherin gained an increase by TGF-beta1 stimulation in SCC-25 (p = 0.007) (Fig. 5 B) and in UPCI-SCC090 cells (Supplementary Figure S4 B, p = 0.04). In FaDu cells TGF-beta1 stimulation also caused increase in N-cadherin gene expression, which was not significant (Supplementary Figure S4 E). The effects of environmental TGF-beta1 and IL-6 did not induce significant changes in HSP-70 gene expression in all three cell lines examined (Supplementary Figure S4 A, D, G). IL-6 did not show significant changes in the investigated cell lines at mRNA level (Supplementary Table S3).

**Fig. 6:**
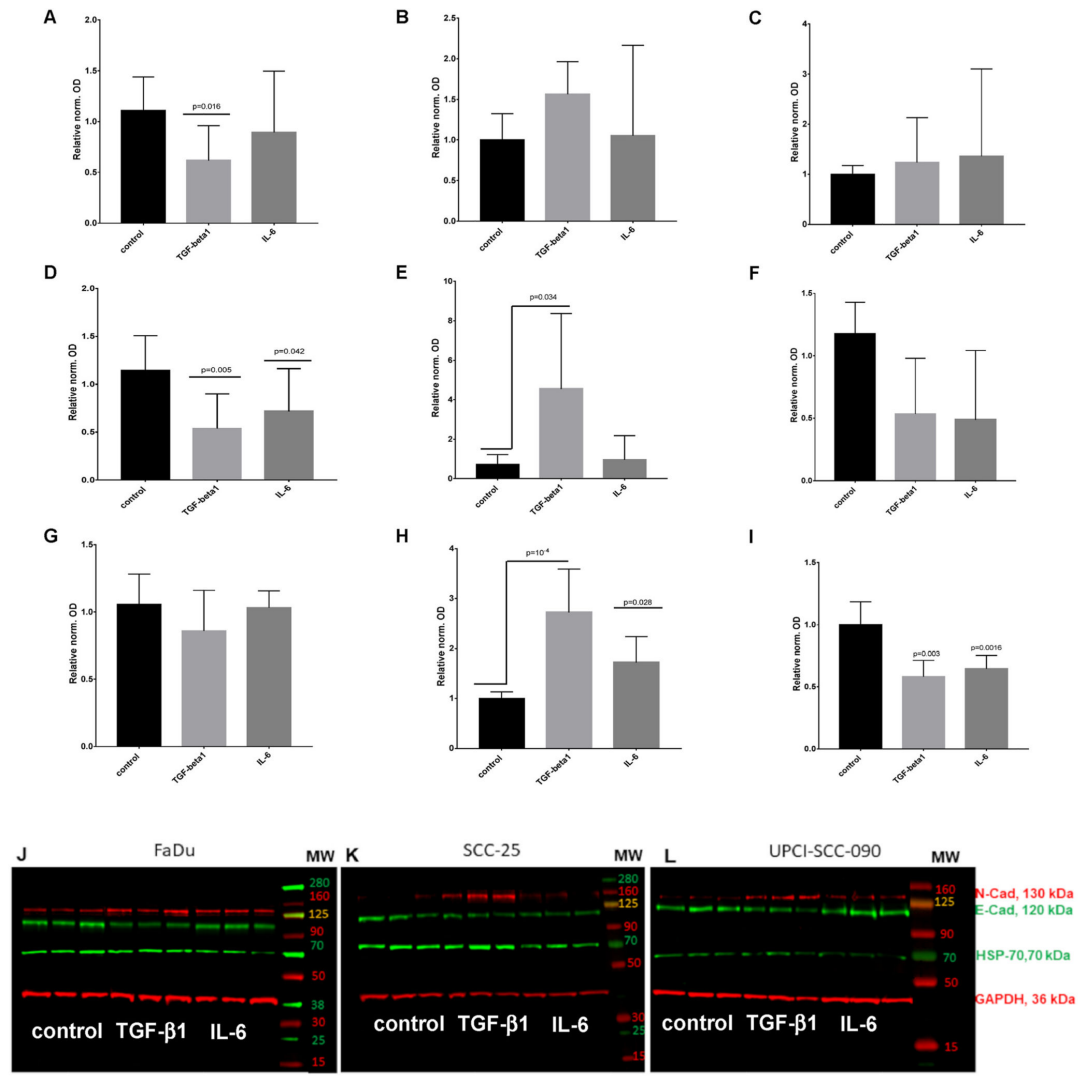
Quantitative analysis of western blots for E-cadherin (A, D, G); N-cadherin (B, E, H) and HSP-70 (C, F, I) in FaDu (A–C), SCC-25 (D–F) and UPCI-SCC-090 (G–I) cells based on 6 repeats (N = 6). Specific bands were detected by near infrared fluorescence. The optical density of the specific bands of interest was normalized with that of GAPDH. In all cases the normalized optical density of the control sample was considered as “1”. Treatment effects are displayed relative to the controls in fold change ± standard error of measurement. Treated conditions were compared with the controls by Dunnett’s multiple comparisons test. Representative images of Western blots in the investigated HNSCC cell lines: FaDu (J), SCC-25 (K), and UPCI-SCC-90 (L). Detected proteins from up to down on the western blots: mesenchymal N-cadherin (molecular weight 130 kDa), epithelial E-cadherin (molecular weight 120 kDa), HSP-70 (molecular weight 70 kDa) and loading control GAPDH (36 kDa). The western blot images represent three repeats of control and treatments with 1 ng/ml TGF-beta1 or with 50 ng/ml IL-6.

The western blot detection showed a significant protein regulation of E-cadherin, N-cadherin, and HSP-70 via TGF-beta1 signalling in HNSCC cell lines. Based on previous reports, our results confirmed the characteristic downshifting of E-cadherin and upregulation of N-cadherin caused by TGF-beta1 pathway that is called, cadherin switch. Nevertheless, the changes at protein level and gene expression of E-cadherin, N-cadherin, and HSP-70, by TGF-beta1 stimulation were different in HNSCC cell lines. In FaDu cells TGF-beta1 treatment induced a significant downregulation of E-cadherin protein (Fig. 6 A (p= 0.016 by Kruskal-Wallis test and Dunnett’s multiple comparison;) and Fig. 6 J) and an upregulation of N-cadherin (Fig. 6 B and J), but this increase was not statistically significant. IL-6 did not induce significant changes in E-cadherin or in N-cadherin protein levels in FaDu cells (Fig. 6A and B, J). HSP-70 was not significantly regulated by TGF-beta 1 or by IL-6 in FaDu and SCC-25 cells (Fig. 6 C, F, J and K). Similarly to FaDu cells, treatment with TGF-beta1 significantly decreased E-cadherin (p = 0.005) (Fig. 6 D and K) and increased N-cadherin protein synthesis (p = 0.034) (Fig. 6 E and K) in SCC-25 cells. Moreover, also IL-6 induced significant decrease in E-cadherin protein levels of SCC-25 cells (p = 0.042) (Fig. 6 D and K), whereas IL-6 did not change the N-cadherin levels significantly (Fig. 6 E and K).

Interestingly, in UPCI-SCC090 cells (HPV-positive model cell line with stable detected KLF4 protein) the E-cadherin protein level was not significantly regulated by TGF-beta1 and IL-6 (Fig. 6 G and L). Although, we recognized a statistically significant upregulation of the mesenchymal component N-cadherin by TGF-beta1 (p = 10 ^–4^) and IL-6 (p = 0.028) treatment (Fig. 6 H and L). These changes were comparable with the mRNA changes described before (Supplementary Table S3). In contrast to FaDu and SCC-25 cells, both, TGF-beta1 and IL-6 treatment induced a significant downregulation of HSP-70 (p = 0.003-TGF-beta 1; p = 0.0016-IL-6) in UPCI-SCC090 cells (Fig. 6 I and L).

### 4.5 KLF4 overexpression regulates the epithelial adhesion system by upregulation of E-cadherin and HSP70 and induces MET in HNSCC tumor cells

**Fig. 7:**
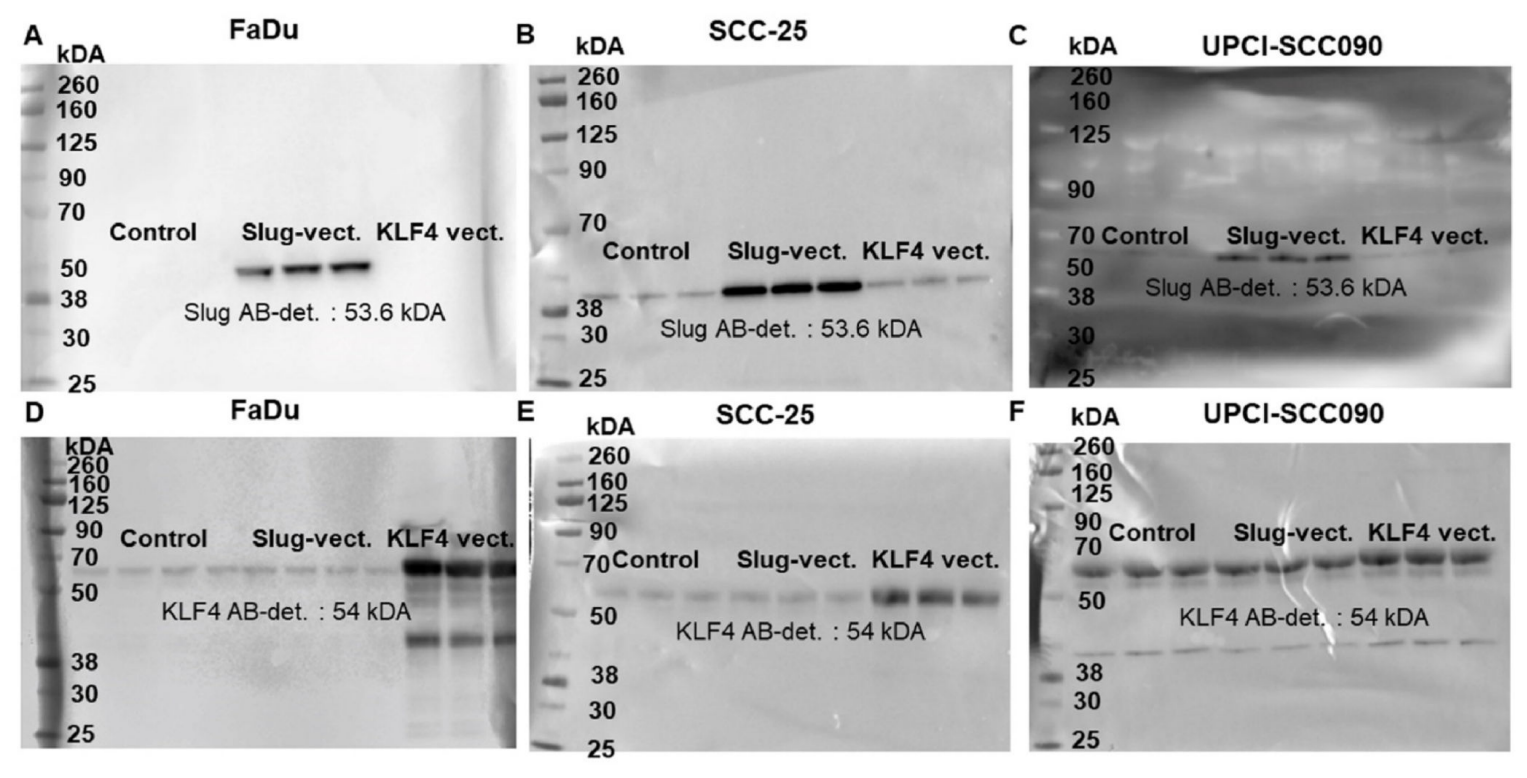
FaDu (A, D), SCC-25 (B, E) and UPCI-SCC090 (C, F) cells were transfected with control, Slug- and KLF4-overexpressing vectors. The protein induction was evaluated by western blot using Slug (A–C) and KLF4 (D-F)-specific antibodies. The immunoblot reactions were detected by horseradish peroxidase-labelled secondary antibodies and chemiluminescent substrate reaction.

We used KLF4 and Slug cloned expression vector to investigate if KLF4 and Slug directly regulated each other and E-cadherin, N-cadherin, HSP-70. At protein level it looks like that Slug and KLF4 did not influence the expression of each other (Fig. 7). In Fig. 7 the protein levels of Slug and KLF4 are displayed after transfection with control, Slug-overexpressing, and KLF4-overexpressing vectors. In all cell lines the overexpression vectors induced a clearly visible increase of the coded proteins’ levels. Interesting was the comparison of the overexpression of Slug and KLF4 in SCC-25 and UPCI-SCC090 cells. SCC-25 cells had significant constitutive baseline protein levels of Slug, which was highly induced by the ectopic overexpression (Fig. 7 B). UPCI-SCC090 cells had low levels of Slug, which was although induced by the ectopic overexpression, but the induced protein levels were far lower than those of SCC-25 cells (Fig. 7 C). On the contrary, KLF4 constitutive baseline protein levels were low in FaDu and SCC-25 cells, and it was highly induced by the ectopic overexpression (Fig. 7 D, E). In UPCI-SCC090 the KLF4 constitutive baseline protein levels were high, which was moderately further induced by the ectopic overexpression (Fig. 7 F).

**Fig. 8:**
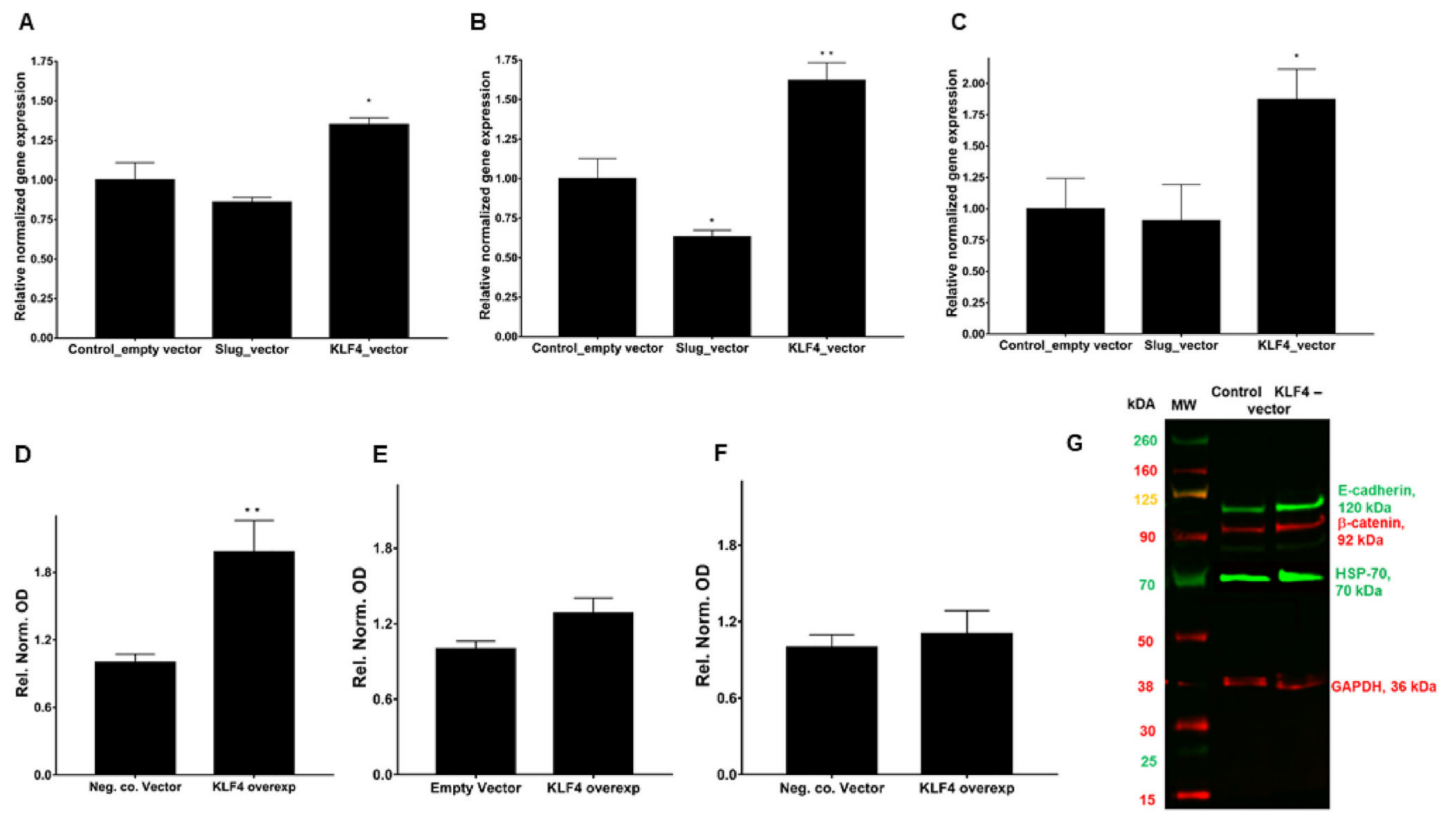
Relative gene expression of E-cadherin (A), β-catenin (B), and HSP-70 (C) in SCC-25 cells after ectopic overexpression of Slug or KLF4. Plasmid containing CMV-promoter without ORF insert was used as control. Slug overexpression showed tendential downregulations, but these were not statistically significant, whereas, KLF4 overexpression significantly upregulated E-cadherin, β-catenin and HSP-70. Protein quantification of E-cadherin (D), β-catenin (E) and HSP-70 (F) after KLF4 overexpression. The significant upregulation of E-cadherin by KLF4 overexpression was also confirmed at protein level (D, G). N = 6 (A–E), N = 8 (F).

The relative gene expression of E-cadherin (Fig. 8 A), β-catenin (Fig. 8 B), and HSP-70 (Fig. 8 C) in SCC-25 cells after ectopic overexpression of Slug or KLF4 was investigated by real-time RT-PCR. Plasmid containing CMV-promoter without ORF insert was used as control. Slug overexpression induced tendential downregulations of E-cadherin, but these were not statistically significant, whereas, KLF4 overexpression significantly upregulated E-cadherin (p = 0.024), β-catenin (p = 0.033) and HSP-70 (p = 0.035) (Fig. 8 A, B, C). At protein level E-cadherin was induced significantly 2 ± 0.7 –times (p = 0.0087 by Mann-Whitney-test) (Fig. 8 D), β-catenin and HSP-70 did not increase significantly by KLF4 vector in SCC-25 cells in repeated transfection experiments (Fig. 8 E-G).

## 5 Discussion

EMT is a product of crosstalk between intrinsic and extrinsic signalling pathways [6,36], regulated by various transcription factors and mainly associated with HNSCC cell invasion and metastasis [23].

Our previous published data indicate a negative correlation between the zink finger-type transcription factor, KLF4, and the EMT-related biomarker Slug in HNSCC. This negative relationship declared, that KLF4 gene expression decreased under the level of normal mucosa, whereas, Slug gene expression significantly increased compared with normal mucosa controls [5].

Based on available references [17,19,31], KLF4 was hypothesized as a positive regulator of E-cadherin, whereas the pro EMT-TF Slug was previously reported to negative regulate both E-cadherin and KLF4 [37].

Loss of E-cadherin in tumor cells enables metastatic dissemination and it’s downregulation has been suggested as an important hallmark of EMT [38–40]. Several mechanisms regulate and negatively affect the functionality of E-cadherin expression: Degradation at protein level, epigenetic regulation and transcriptional changes within a decrease of E-cadherin gene expression were shown to impede the maintenance of E-cadherin. Moreover, the presence of HSP-70 was shown to play a key role for the stabilization of the cadherin/catenin complex. Cancer cells in the absence of HSP-70 were accompanied by the deregulation and the re-localization of E-cadherin from the cell-surface into the cytosol; subsequently it has been demonstrated that this dysfunction releases β-catenin from the cell-cell junctions [35].

Here we found a negative relationship between Slug and E-cadherin gene expression at mRNA level and as already published by our clinic, Slug further negative correlated with E-cadherin and beta-catenin in quantitative immunohistochemistry at protein level [27].

Subbalaskshmi et al. described a major opposing relationship between Slug (EMT-inducer) and KLF4 based on mathematical modelling and transcriptomic data analysis, nevertheless, this hypothesis was not evidenced by them experimentally. Compatible to our results, the authors identified a negative correlation of KLF4 with EMT-related TFs and with transcriptomic based EMT scoring metrics in the Cancer Cell Line Encyclopedia (CCLE) cell lines. Overall, KLF4 was highlighted as an important MET-inducing transcription factor by inhibiting crucial EMT-TFs directly and/or indirectly [18].

In our study, we were interested to test the hypothesis that Slug as a major EMT-transcription factor in HNSCC promotes EMT by suppressing E-cadherin and cadherin-based epithelial adhesion, whereas, KLF4, which promotes the epithelial state in opposite, presents itself as a MET-transcription factor and induces epithelial phenotype. We tested this hypothesis at mRNA and protein levels in HPV-positive and negative HNSCC tissue samples and in three HNSCC model cell lines. Slug, KLF4 and E-cadherin mRNA was detected by RT-PCR, and further examined in HNSCC specimens using multichannel immunofluorescence microscopy.

First, we found that KLF4 and E-cadherin gene expression showed a high significant positive correlation (p<10^–4^) in our clinical HNSCC RNA data bank and in the HNSCC databank of TCGA (p<10-^3^) (Fig. 1 A–B). In head and neck cancer the regulation of KLF4 gene expression was further influenced by human papilloma virus. Our results revealed increased KLF4 and E-cadherin gene expression in HPV-positive HNSCC. Using quantitative immunofluorescence we confirmed a higher staining intensity (Fig. 2 C-D-E-F) and an enhanced percentage of cells stained with KLF4 (Supplementary Figure S1 A) in HPV-positive HNSCC than in HPV-negative HNSCC. These results further revealed that HPV-virus products might be activators and stabilizers of KLF4 protein in real life pathologies. This finding was further underwired by western blot analysis in HPV-positive UPCI-SCC090 cells, which had high stable baseline constitutive KLF4 levels (Figs. 3 A, 9 F). In consequence, TGF-beta1 was not able to trigger the cadherin switch in UPCI-SCC090 cells, although N-cadherin was induced, but E-cadherin was not reduced, due to the stable KLF4 levels, which could be the consequence of the stabilization effect of the HPV-background.

Our results support previous findings that KLF4 levels and activity were increased in HPV-positive cells [20] in *vitro* and in HNSCC tumor tissue samples. The HPV-positive model cell line UPCI-SCC090 showed a stabilized and consequently high KLF4 protein expression which might be due to post-transcriptional and post-translational mechanisms. According to Gunasekharan et al., E7 gene expression mediated the suppression of cellular miR-145 at post transcriptional level and presence of E6 contributed to the inhibition of KLF4 phosphorylation at post-translational level. This could be also observed in UPCI-SCC090 cells, where the pCMV-promoter-induced overexpression had a minor effect on KLF4 protein levels, which were constitutively at a high level. There are several stabilization mechanisms at protein level, which are posttranslational, all of them are effective and modify the transcription effects of pCMV-induced ectopic gene expression.

In a future study, we will further investigate if KLF4, after ectopic expression under the control of the strong CMV-promoter, only induces the epithelial differentiation-associated functions, or it is also responsible for increased proliferation, which is clinically relevant property of epithelial tumor cells. Preliminary results (Supplementary Figure S5) suggest that transfection with KLF4 overexpressing vector increase cell proliferation at approximately 30% over that of the control vector transfected untreated cells. In consistence with the mathematical-based publication of Subbalaskshmi et al. [18], our findings revealed that KLF4 is strongly engaged with epithelial phenotype which is the proliferating form of HNSCC tumor cells [41]. The effect of KLF4 is tissue and context dependent. In progressed HNSCC tumor according to Tai et al. it is related with poor prognosis, which might be explained by the re-activated MET process with proliferation of therapy resistant tumor cells [18].

There are very few proven strategies to induce MET in culture. As published before [18,42,43], KLF4 might also act as a potential inducer of MET. Through mechanisms that are not completely understood, binding of KLF4 allows the chromatin to “breathe” thus allowing binding of other transcription factors to initiate gene transcription [44]. KLF4 was shown to bind to the E-cadherin promoter directly [17,45], and to act as a transcriptional repressor of genes critical for EMT, including SLUG and JNK1 [31].

This work provided evidence, that the positive gene induction effect of KLF4 at mRNA level is essential for switching on of the epithelial adhesion system during re-epithelialization (MET) after EMT, where, in concert, E-cadherin and beta-catenin are upregulated. In contrast, Kasioumi et al. described the role of stabilization factors at protein level, such as HSP-70, for the primarily maintenance of the epithelial character and phenotype [35]. Even significant induction of mesenchymal adhesion molecule as N-cadherin might co-exist with stable E-cadherin in cells with stabilized KLF4 (Fig. 7 L).

## 6 Conclusion

Based on our experimental data we hereby propose KLF4 as a potential MET-inducer and transcriptional activator of the epithelial adhesion system in HNSCC. We identified a significant positive correlation of KLF4 and E-cadherin gene expression in our HNSCC RNA data bank and in the HNSCC data bank of The Cancer Genome Atlas (TCGA). Moreover, we confirmed the stabilization effects of HPV-virus transcripts on KLF4 in UPCI-SCC090 cells and in HPV-positive tumor tissue samples. Our study suggests, that TGF-beta1 induced EMT contributed to the induction of Slug and increased levels of mesenchymal N-cadherin, whereas, HSP-70 and the epithelial markers KLF4 and E-cadherin were downregulated in cells where KLF4 was not stabilized by epigenetic factors. Furthermore, KLF4 overexpression was associated with restoration of E-cadherin and β-catenin levels, upregulation of other critical epithelial genes, such as HSP70, and the induction of cell proliferation during MET.

## Supplementary Material

Table

Tables, Figures

## Figures and Tables

**Table 1:** Utilized primary antibodies for immunohistochemistry and immunofluorescence staining.

Antibody	Isotype	catalogue number	manufacturer	dilution
pan-cytokeratin	Mouse IgG1	760–2595	Roche Ventana, Mannheim, Germany	prediluted
anti-KLF4	Rabbit IgG	ab215036	Abcam, Cambridge, UK	1:800
anti-Slug	Rabbit IgG	9585	Cell Signalling, Danvers, MA, USA	1:30
anti-E-cadherin	Mouse IgG2a	790–4497	Roche Ventana, Mannheim, Germany	prediluted
anti-HSP70	Mouse IgG2b	818,101	Biolegend, California, USA	1:100
Isotype-control mouse	Mouse IgG1	11-632-C100	Exbio, Prague, Czech Republic	1:100
Isotypecontrol mouse	Mouse IgM	11-803-C100	Exbio, Prague, Czech Republic	1:100
Isotypecontrol rabbit	Mouse IgG	02–6102	Invitrogen Life Technologies, Darmstadt, Germany	1:400

Primary antibodies and isotype controls were used at maximal 10 μg/ml concentration. The isotype controls were used at the maximal concentration of the primary antibodies.

**Table 2:** Overview of utilized antibodies for Western-blot.

Antibody	Isotyp	catalogue number	kDa	manufacturer	dilution
anti-N-cadherin	IgG1	61 09 20	130	BD/Biosciences	1:1000
anti-E-cadherin	IgG2a	610,181	120	BD/Biosciences	1:2500
anti-β-catenin	IgG1	61 01 53	92	BD/Biosciences	1:4000
anti-HSP-70	IgG2b	818,101	70	Biolegend, California, USA	1:200
anti-Slug	Rabbit IgG	9585	30	Cell Signaling, Danvers, MA, USA	1:1000
anti-KLF4	Rabbit IgG	ab215036	54	Abcam, Cambridge, UK	1:1000
anti-GAPDH	IgG1	ab8245	36	Abcam, Cambridge, UK	1:5000
Fluorochrome secondary antibody					
αmouse IgG2a 800 CW	IgG2a	926-32351		LiCor Bioscience, Germany	1:5000
α mouse IgG1 680LT	IgG1	926-68050		LiCor Bioscience, Germany	1:10,000
α rabbit IgG 800CW	IgG	926-32213		LiCor Bioscience, Germany	1:5000
α mouse IgG IR800	IgG	AC2135		Azure	1:2500
Peroxidase secondary antibody					
α mouse IgG HRP	IgG	31,430		Pierce, ThermoFisher Scientific	1:10,000
α rabbit IgG HRP	IgG	31,460		Pierce, ThermoFisher Scientific	1:10,000

## Abbreviations

CT: Threshold Cycle
CCLE: Cancer Cell Line Encyclopedia
EMT: Epithelial-to-mesenchymal transition
GAPDH: Glycerin Aldehyde Dehydrogenase
HNSCC: Head and neck squamous cell carcinoma
HPV: Human papilloma virus
IL-6: Interleukin-6
KLF4: Krüppel-like-factor-4
MET: mesenchymal-to-epithelial transition
ORF: open reading frame
SLUG: Transcription Factor Slug
TCGA: Cancer Genome Atlas database
TF: Transcription Factor
TGF-beta1: Transforming-growth-factor-beta-1
UPPP: Uvulopalatopharyngoplasty
