# Supplementary material for: EMT-related transcription factors and protein stabilization mechanisms involvement in cadherin switch of head and neck squamous cell carcinoma
Source: Exp Cell Res. Author manuscript; Available in PMC 2022 Dec 18. (PMC7613960; doi:10.1016/j.yexcr.2022.113084)
Supplement: Tables, Figures [file EMS158130-supplement-Tables__Figures.docx]

**Supplementary Material:**

**Table S1. Study population**

| **sex** | male | 64 | 90% |
| --- | --- | --- | --- |
|  | female | 5 | 7% |
|  | missing | 2 | 3% |
| **age** | <=50 | 4 | 6% |
|  | 51–60 | 15 | 21% |
|  | 61–70 | 29 | 41% |
|  | 71–80 | 15 | 21% |
|  | >80 | 6 | 9% |
|  | missing | 2 | 3% |
| **ASA-Score** | ASA I/II | 41 | 58% |
|  | ASA III/IV | 22 | 31% |
|  | missing | 8 | 11% |
| **tumor site** | lips/oral cavity | 6 | 9% |
|  | oropharynx | 36 | 51% |
|  | hypopharynx | 14 | 20% |
|  | Larynx | 13 | 18% |
|  | others | 2 | 3% |
| **histology** | squamous cell carcinoma | 68 | 96% |
|  | lymphoepithelial carcinoma | 1 | 1% |
|  | missing | 2 | 3% |
| **HPV/tumor localization** | P16-positive oropharynx SCC | 20 | 28% |
|  | P16-negative oropharynx SCC | 16 | 23% |
|  | P16-positive larynx SCC | 1 | 1% |
|  | P16-negative larynx SCC | 12 | 17% |
|  | P16-negative SCC of lips/oral cavity | 5 | 7% |
|  | P16-negative SCC of hypopharynx | 13 | 18% |
| **UICC stage** | stage 1 | 5 | 7% |
|  | stage 2 | 5 | 7% |
|  | stage 3 | 16 | 23% |
|  | stage 4a | 32 | 45% |
|  | stage 4b | 5 | 7% |
|  | stage 4c | 5 | 7% |
|  | missing | 3 | 4% |
| **P16 status** | negative (<70%) | 46 | 65% |
|  | positive (>=70%) | 23 | 32% |
|  | missing | 2 | 3% |
| **P53 status** | regular | 31 | 44% |
|  | irregular | 38 | 53% |
|  | missing | 2 | 3% |
| **treatment modality** | surgery | 11 | 15% |
|  | surgery & RT | 7 | 10% |
|  | surgery & RCHT/RIT | 1 | 1% |
|  | RCHT/RIT | 27 | 38% |
|  | RT | 1 | 1% |
|  | missing | 24 | 34% |

Table 1a. Patient characteristics whose samples were used for RNA analysis.

| **sex** | male | 52 | 90% |
| --- | --- | --- | --- |
|  | female | 6 | 10% |
| **age** | <=50 | 4 | 7% |
|  | 51–60 | 11 | 19% |
|  | 61–70 | 21 | 36% |
|  | 71–80 | 16 | 28% |
|  | >80 | 6 | 10% |
| **ASA-Score** | ASA I/II | 32 | 56% |
|  | ASA III/IV | 25 | 44% |
| **tumor site** | lips/oral cavity | 10 | 17% |
|  | oropharynx | 29 | 50% |
|  | hypopharynx | 8 | 14% |
|  | Larynx | 10 | 17% |
|  | others | 1 | 2% |
| **histology** | squamous cell carcinoma | 58 | 100% |
| **HPV/tumor localization** | P16-positive oropharynx SCC | 13 | 22% |
|  | P16-negative oropharynx SCC | 16 | 28% |
|  | P16-positive larynx SCC | 0 | 0% |
|  | P16-negative larynx SCC | 10 | 17% |
|  | P16-negative SCC of lips/oral cavity | 10 | 17% |
|  | P16-negative SCC of hypopharynx | 8 | 17% |
| **UICC stage** | stage 1 | 5 | 9% |
|  | stage 2 | 3 | 5% |
|  | stage 3 | 13 | 22% |
|  | stage 4a | 26 | 45% |
|  | stage 4b | 5 | 9% |
|  | stage 4c | 6 | 10% |
| **P16 status** | negative (<70%) | 45 | 78% |
|  | positive (>=70%) | 13 | 22% |
| **P53 status** | regular | 18 | 31% |
|  | irregular | 40 | 69% |
| **treatment modality** | surgery | 13 | 22% |
|  | surgery & RT | 15 | 26% |
|  | RCHT/RIT | 23 | 40% |
|  | RT | 4 | 7% |
|  | Best supportive care | 3 | 5% |

Table 1b. Patient characteristics whose samples were used for immunohistochemistry.

**Table S2. List of used PCR primers in the Study.**

| **Gene** | **Forward primer** | **Reverse primer** |
| --- | --- | --- |
| GAPDH | TGCACCACCAACTGCTTAGC | GGCATGGACTGTGGTCATGAG |
| E-cadherin [CDH1] | CGAGAGCTACACGTTCACGG | GGGTGTCGAGGGAAAAATAGG |
| HSP70 | TTGGACGGAAATTCGAGGATG | AAATAGGCCGGGACCGTTATG |
| N-cadherin [CDH2] | AGCCAACCTTAACTGAGGAGT | GGCAAGTTGATTGGAGGGATG |
| Beta-catenin [CTNNB1] | AAAGCGGCTGTTAGTCACTGG | CGAGTCATTGCATACTGTCCAT |
| KLF4 | TCCAAAGAAGAAGGATCTCGGCCA | AACGTGGAGAAAGATGGGAGC A |
| SNAI2 | ACATAAGCAGCTGCACTGCG | ATGGGTCTGCAGATGAGCCC |

**Figure S1.** Percentages of cells in the cancer cell nest stained with (**A**) KLF4 (p=0.04, with Student´s t-test, the data were normal distributed) and (**B**) E-cadherin (p=0.8 with Mann-Whitney U-test, the data were not normal distributed), both in relation to HPV genetical background. Number of cases included: **A**: HPV^-^:42, HPV^+^:14; **B**: HPV^-^:32, HPV^+^:9

**Figure S2.** Correlation between Slug and E-cadherin relative staining intensity. Regression line at 95% confidence interval. (N=33, negative correlation Spearman r=-0.34; p=0.05).


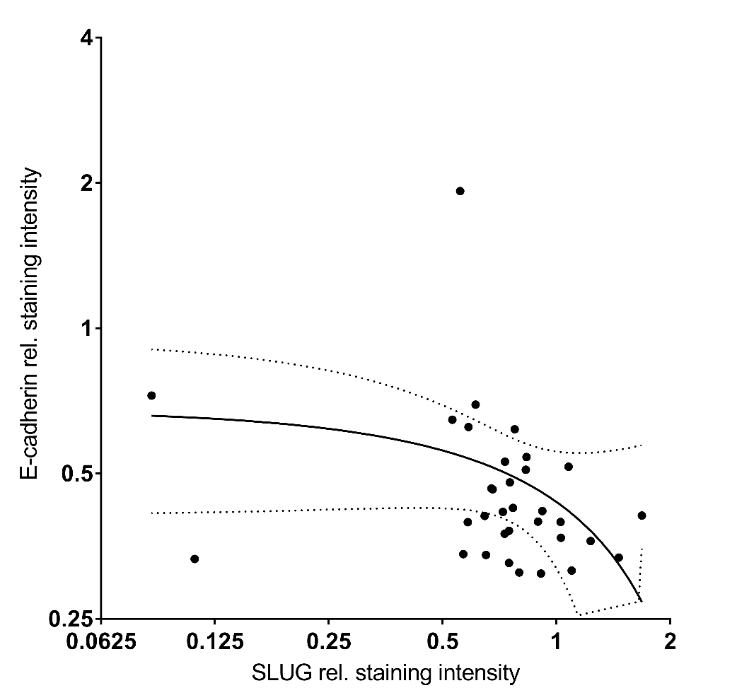


**
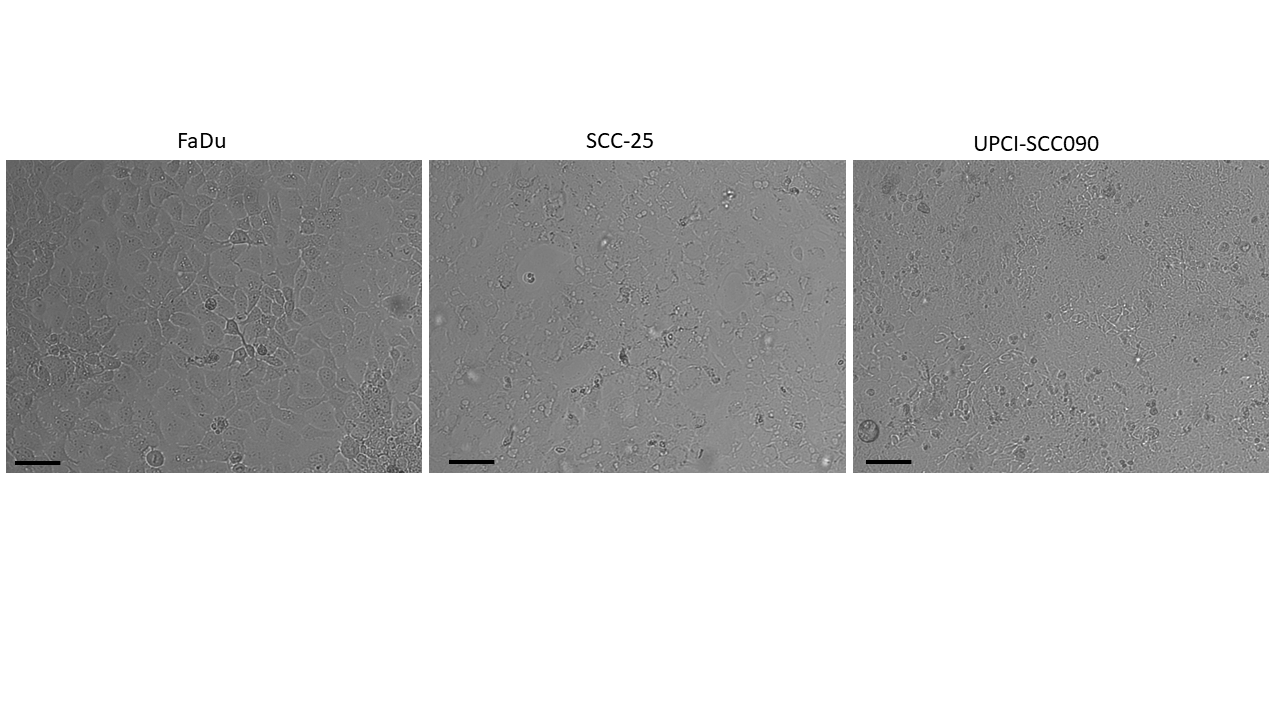
Figure S3.** Phase contrast images of confluent cultures of the investigated HNSCC cell lines: FaDu (confluent at day 5 of culture), SCC-25 and UPCI-SCC090 (confluent at day 8-10 of culture). Bars: 100 µm.

**Figure S4.** Relative gene expression analysis of HSP-70, N-cadherin and E-cadherin by TGF-beta1 and IL-6 treatment in UPCI-SCC090 cells (**A-C**) and FaDu cells (**D-F**). Changes in HSP-70 by TGF-beta1 and IL-6 stimulation in SCC-25 cells (**G**). Details of the statistical analysis are listed in Supplementary Table S3.

**
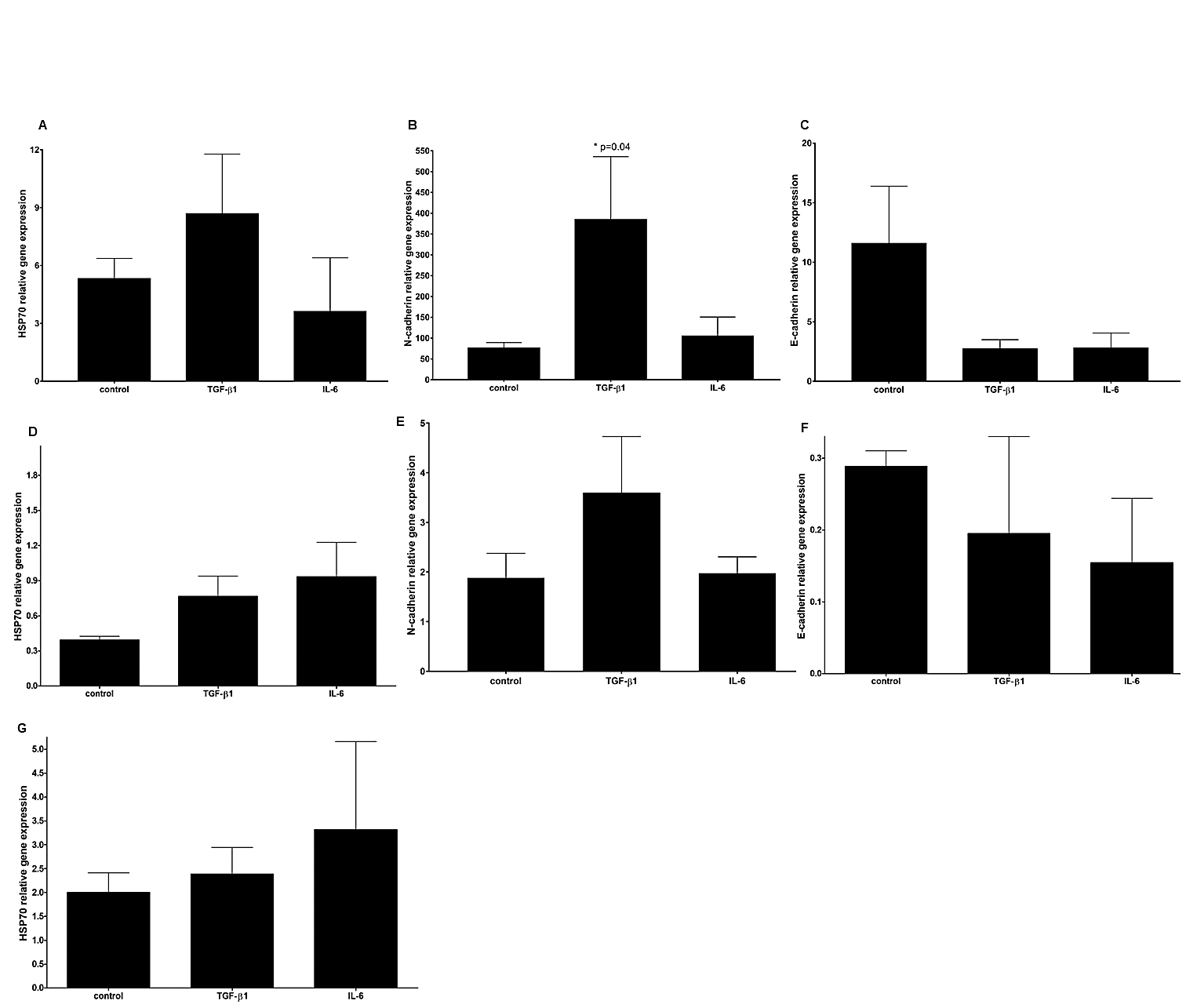
**

**Figure S5. KLF4 ectopic overexpression in EMT cells induces cell proliferation.** Five days 1 ng/ml TGF-beta1 treatment was followed by transfection with control pCMV3 vector or with pCMV3-KLF4. The cell numbers were counted 48 hours after the transfection. TGF-beta1 reduced the cell counts to 50% of the control cells. KLF4 overexpression significantly (p= 6 * 10^-4^ by Mann Whitney test) increased the cell counts. If TGF-beta1 treatment was followed by pCMV3-KLF4 transfection, the cell counts increased significantly (p=0.038 by unpaired t-test). (N=18).
